# Allergen specificity is relevant for immunotherapy prescription in polysensitised children

**DOI:** 10.1186/1824-7288-38-50

**Published:** 2012-09-21

**Authors:** Giorgio Ciprandi, Cristoforo Incorvaia, Paola Puccinelli, Ilaria Dell’Albani, Franco Frati

**Affiliations:** 1 IRCCS-Azienda Ospedaliera Universitaria San Martino di Genova, Viale Benedetto XV 6, 16132, Genoa, Italy; 2 Istituti Clinici di Perfezionamento, Milan, Italy; 3 Scientific Department, Stallergenes, Milan, Italy

**Keywords:** Polysensitization, Prescription, Allergen-specific immunotherapy

## Abstract

The sensitization to more allergens, such as polysenitization, is becoming a frequent characteristic of allergic patients, since the childhood. However, this phenomenon is considered an obstacle to prescribe immunotherapy by many doctors. This study investigated the relevance of polysensitization in a cohort of allergic children and evaluated the number of allergen extracts prescribed for these children. There are allergens that are frequent, but not prescribed. This issue should be matter of adequate debate for Italian paediatricians.

## 

Sensitization is the phenomenon characterized by the production of allergen-specific IgE. It is the *sine qua non* requirement for allergy. However, true allergy diagnosis is based on the documented demonstration of a cause-effect relationship between the exposure to sensitizing allergen and symptom occurrence. On the other hand, most of allergic children are sensitized to more allergens, such as polisesnitized [1]. Thus, the presence of polysensitization may represent an obstacle for prescribing allergen-specific immunotherapy, which is the possible strategy to cure respiratory allergy. Therefore, polysensitization constitutes a relevant problem in the management of allergy. In this regard, Calderon and colleagues have recently revised the issue concerning the immunotherapy strategy in polysensitized patients [2].

We believe that many aspects may be discussed about this relevant topic. We would like to point out some considerations. Firstly, we conducted some real life trials both in adults and in children. In particular, there is a paucity of studies concerning the pediatric age on this topic. We initially analyzed the data of 139 children enrolled in 9 Italian allergy centers [3]. The findings showed that most (about 60%) children had persistent allergic rhinitis of moderate-severe grade and half of them had asthma associated with rhinitis. Further, we evaluated 51 polysensitized children treated with sublingual IT (SLIT) by Staloral 300 IR (Stallergenes, Antony, France) for 1 year [4]. SLIT significantly reduced the severity of rhinitis as well as of symptoms, including ocular, nasal, and bronchial complaints. Therefore, this study did confirm that polysensitization was not an obstacle to prescribe IT, and particularly in children.

Another important issue is represented by the discrepancy between the prevalence of specific sensitization and the relative prescription. We believe that this topic should be clinically relevant in the common practice. For this reason, we carefully analyzed the pediatric (0-17 years) patients. Interesting findings were found in this pediatric population (Figure 1), the most frequent allergens causing sensitization were grass pollen (88%), mites (41%), and Betulaceae (39%). Some differences in comparison with adults exist: mite sensitization always corresponds to IT prescription, as well as ¾ of grass pollen sensitizations. On the contrary, all other pollen sensitizations (with the exception for Parietaria) never were lead to IT, as well as cat and dog sensitization. These data are clinically relevant and should be matter of debate. Moreover, we believe that the correct attitude to adopt in polysensitized children should be based on a careful history and on the analysis of potential causal allergens [5]. We retain that to renounce to immunotherapy is too simply and reductive.

**Figure 1:**
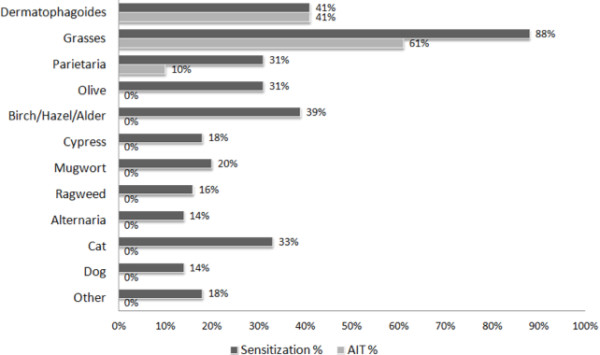
Frequencies of allergen sensitizations (dark bar) and allergen prescription for immunotherapy: AIT (light bar) in polysensitized children.

## Conclusion

We would like to underline that the issue concerning the discrepancy between sensitization and IT prescription should be more carefully considered and evaluated in future studies. Moreover, the use of the Component Resolved Diagnosis could improve the capacity of choosing allergens for IT. The challenge for the allergists continues, but more tools will be available soon.

## Competing interests

PP, IDA, and FF are employers of Stallergenes Italia.

GC and CI are consultant of Stallergenes Italia.

Stallergenes Italia funded the study.

## Authors’ contribution

All authors contributed in realizing this Letter. All authors read and approved the final manuscript.
